# Stammering

**Published:** 1891-11

**Authors:** 


### STAMMERING.

The causes of this defect, and the appropriate methods of dealing with it, have been so often discussed it is needless to seek for etiological conditions in the organs of speech themselves, or even in the nerves associated with them. The stammerer, if his mind is at ease, does not stammer. It is in the presence of circumstances, varying in different cases, which to him suggest some imaginary difficulty that his impediment becomes apparent. Founded upon some original shyness, it may be, in addressing a teacher there is a not unnatural delay in utterance. Concurrently with this comes a feeling that he must speak. Intelligence and will together urge him to do so ; the purpose is met by his conscious unreadiness, and the consequence is the marred result with which he himself and his companions are painfully familiar. His impediment therefore, is imaginary. The remedies appropriate to his condition, if somewhat slow in operation, are not far to seek. They consist essentially in a change, a disentanglement of his perverted mental energies. All mental shock is to be strictly excluded. The habit cannot be cured by order. The pupil must be approached with tact, and habitually addressed in a quiet, slow, and deliberate manner. His imitative instinct will copy the method, and fluency will usually succeed the faculty of correct utterance thus engendered. When we reflect upon the frequently high intelligence of stammering children, the drawback imposed upon their education by this unfortunate habit, and its equally hurtful influence upon their usefulness as adults, we cannot too strongly impress the necessity of its early and methodical treatment.

